# Supplementary material for: Seminoma and Embryonal Carcinoma Footprints Identified by Analysis of Integrated Genome-Wide Epigenetic and Expression Profiles of Germ Cell Cancer Cell Lines
Source: PLoS One. 2014 Jun 2;9(6):e98330. doi: 10.1371/journal.pone.0098330 (PMC4041891; doi:10.1371/journal.pone.0098330)
Supplement: File S1 — ZIP file containing DMRforPairs output for significant regions. Please start from the html files. (ZIP) [file pone.0098330.s008.zip › figures/34002.pdf]

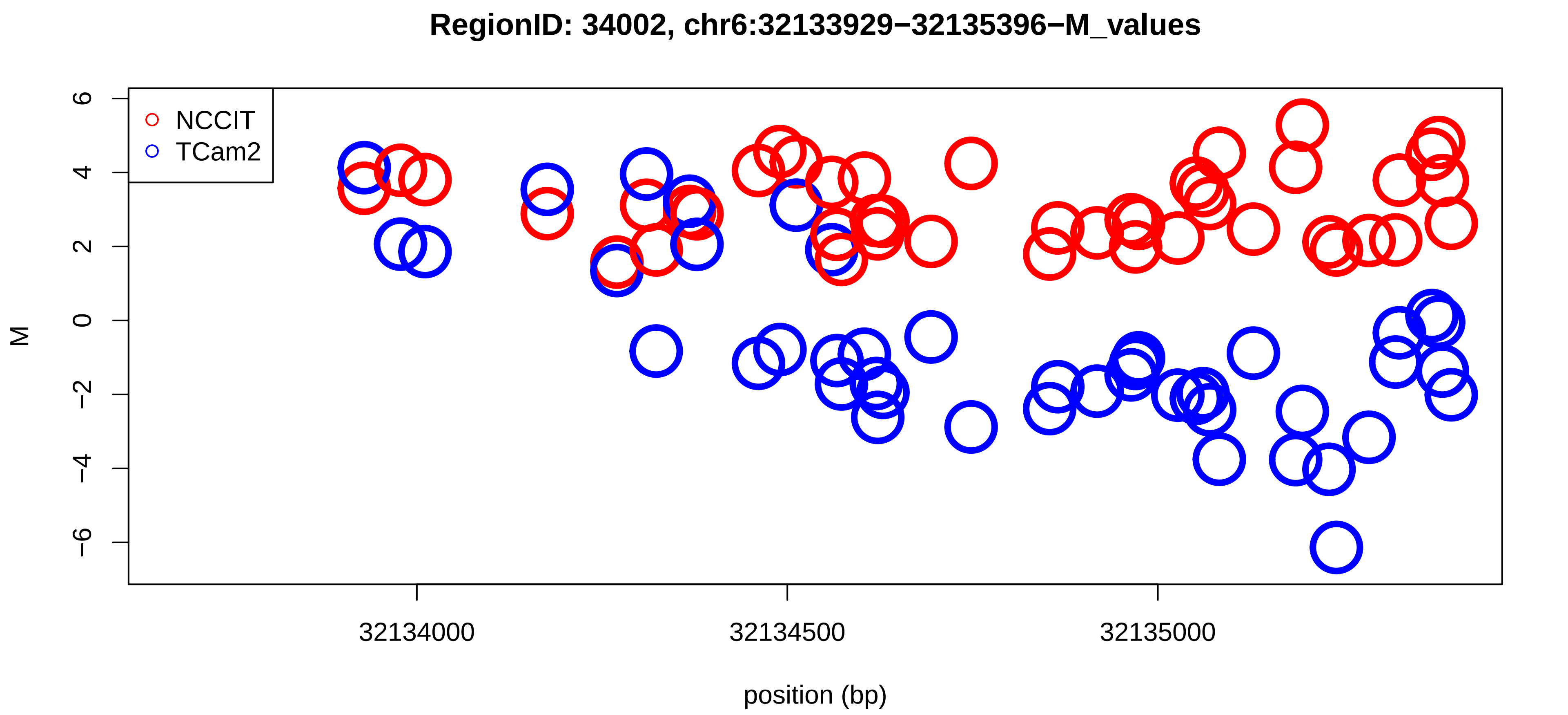
RegionID: 34002, chr6:32133929−32135396−M_values
NCCIT
TCam2
M
6
4
2
0
−2
−4
−6
32134000
32134500
32135000
position (bp)

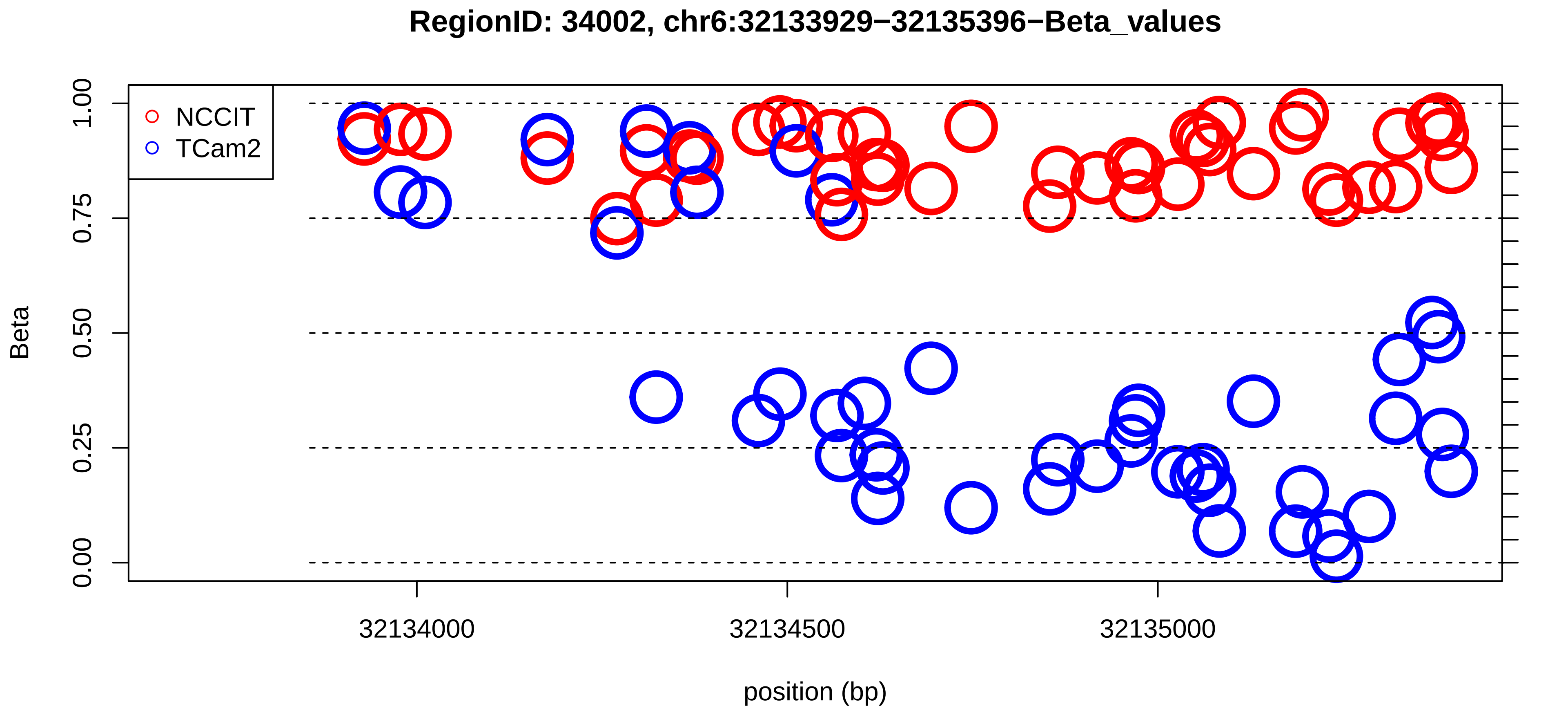
RegionID: 34002, chr6:32133929−32135396−Beta_values
NCCIT
TCam2
Beta
1.00
0.75
0.50
0.25
0.00
32134000
32134500
32135000
position (bp)

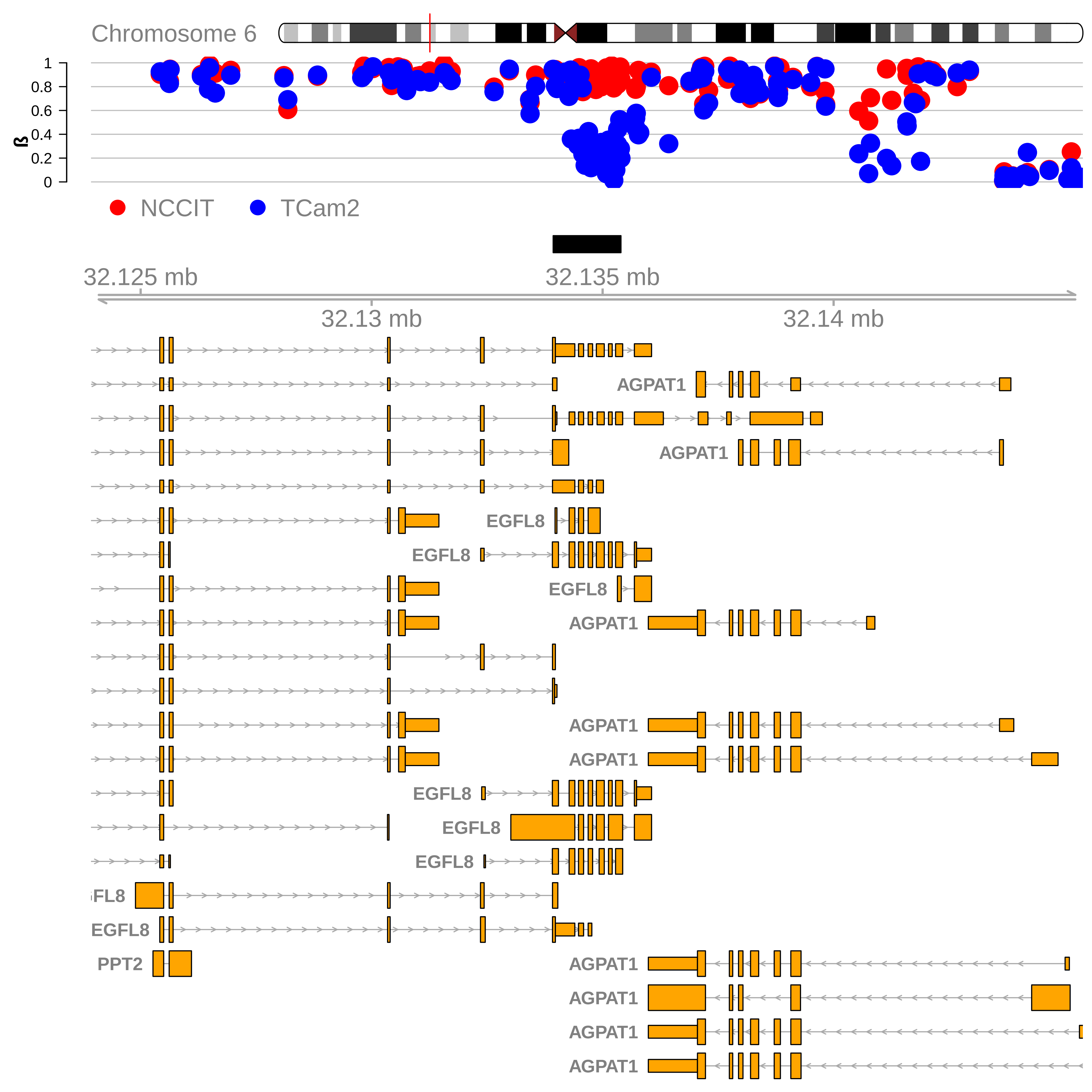

Chromosome 6
ß
1
0.8
0.6
0.4
0.2
0
NCCIT
TCam2
32.125 mb
32.135 mb
32.13 mb
32.14 mb
AGPAT1
AGPAT1
EGFL8
EGFL8
EGFL8
AGPAT1
AGPAT1
AGPAT1
EGFL8
EGFL8
EGFL8
EGFL8
PPT2
AGPAT1
AGPAT1
AGPAT1
AGPAT1
